# The impact of long term institutional collaboration in surgical training on trauma care in Malawi

**DOI:** 10.1186/s40064-016-2050-7

**Published:** 2016-04-05

**Authors:** Sven Young, Leonard Banza, Nyengo Mkandawire

**Affiliations:** Department of Surgery, Kamuzu Central Hospital, P.O. Box 149, Lilongwe, Malawi; Department of Surgery, College of Medicine, University of Malawi, Blantyre, Malawi; Department of Orthopaedic Surgery, Haukeland University Hospital, 5021 Bergen, Norway; Department of Surgery, Queen Elizabeth Central Hospital, Blantyre, Malawi; School of Medicine, Flinders University, Adelaide, SA Australia

**Keywords:** International institutional collaboration, Trauma care, Low-income countries, Training of surgeons, Amputations

## Abstract

**Background:**

Attempts to address the huge, and unmet, need for surgical services in Africa by training surgical specialists in well established training programmes in high-income countries have resulted in brain drain, as most trainees do not return home on completion of training for various reasons. Local postgraduate training is key to retaining specialists in their home countries. International institutional collaborations have enabled Kamuzu Central Hospital (KCH) in Lilongwe, Malawi, to start training their own surgical specialists from 2009.

**Results and discussion:**

The direct impact of this has been an increase in Malawian staff from none at all to 12 medical doctors in 2014 in addition to increased foreign faculty. We have also seen improved quality of care as illustrated by a clear reduction in the amputation rate after trauma at KCH, from nearly every fourth orthopaedic operation being an amputation in 2008 to only 4 % in 2014. Over the years the training program at KCH has, with the help from its international partners, merged with the College of Medicine in Blantyre, Malawi, into a national training programme for surgery.

**Conclusions:**

Our experiences from this on-going international institutional collaboration to increase the capacity for training surgeons in Malawi show that long-term institutional collaboration in the training of surgeons in low-income countries can be done as a sustainable and up-scalable model with great potential to reduce mortality and prevent disability in young people. Despite the obvious and necessary focus on the rural poor in low-income countries, stakeholders must start to see the value of strengthening teaching hospitals to sustainably meet the growing burden of trauma and surgical disease.

**Methods:**

Annual operating data from Kamuzu Central Hospital’s Main Operating Theatre log book for the years 2008–2014 was collected. Observed annual numbers were presented as graphs for easy visualization. Linear regression curve estimations were calculated and plotted as trend lines on the graphs.

## Background

97 % (!) of all deaths in young people aged 10–24 years occur in low and middle income countries (LMICs) with Road Traffic injuries (RTIs) as the single most frequent cause of death (Patton et al. 2009). The global burden of RTIs is growing rapidly and the WHO predicts RTIs will be the fifth most frequent cause of death over all by 2030 (WHO 2009). RTIs disproportionately affect poor people (Peden 2004). In Malawi, in southern Africa, two thirds of all people that die on the roads are pedestrians and cyclists (WHO 2015). Most of the rest die using unsafe public transport. For every RTI death there are 20–50 survivors needing treatment for injuries sustained on the road (Peden 2004). In Uganda 3 times as many as die end up with permanent disability (Kobusingye et al. 2001). This increasing burden of trauma related surgical disease further worsens the unmet surgical need, as Africa only has 3 % of the World’s health workers (Crisp and Chen 2014) and only utilizes 1 % of global health expenditure. With an estimated population of 16 million people (World Bank 2013), Malawi has only four orthopaedic surgeons working full time in government hospitals. In comparison Norway has more than 500 times as many orthopaedic surgeons per population as Malawi. Despite what many global health politicians think, surgery has been shown to be as cost effective as many of the adopted public health interventions for communicable diseases and maternal health, and as safe as in high-income countries (Gosselin et al. 2009; Grimes et al. 2014).

**Fig. 1:**
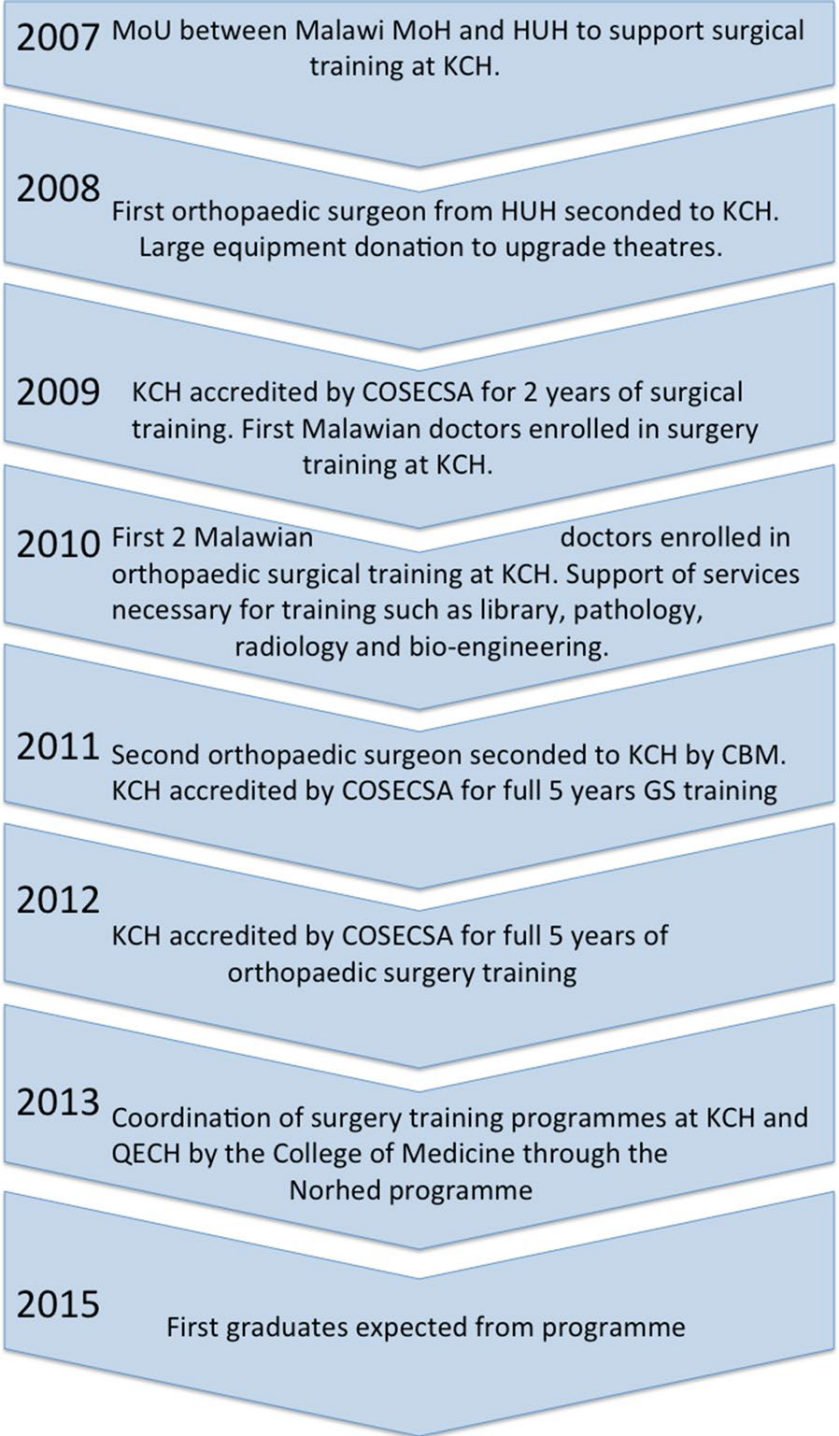
Simplified timeline of the institutional collaboration for surgical training in orthopaedic surgery at Kamuzu Central Hospital

Attempts to address the huge, and unmet, need for surgical services in Africa by training surgical specialists in well established training programmes in high-income countries have resulted in brain drain, as most trainees do not return home on completion of training for various reasons. Local postgraduate training is key to retaining specialists in their home countries (Hagander et al. 2013). Collaboration with Haukeland University Hospital (HUH) in Bergen, Norway, started in 2007 and enabled Kamuzu Central Hospital (KCH) in Lilongwe, Malawi, to start training their own surgical specialists from 2009. From 2010 the University of North Carolina (UNC) joined forces with the other stakeholders to strengthen the programme further. A timeline of the collaboration can be seen in Fig. 1. We wanted to look at how this international institutional collaboration has impacted service delivery in orthopaedic trauma care at KCH with focus on the rate of amputations, and discuss how best to move forward in order to scale up achievements.

## Results and discussion

From having only four foreign general surgeons for a catchment population of 5.5 million people in 2007, KCH now also has 2 seconded orthopaedic surgeons, eight young Malawian registrars training in general surgery, one in urology, one in neurosurgery and two in orthopaedic surgery. This has more than tripled the number of surgical staff, doubled the number of annual operations done and improved the quality of the surgical services considerably (Kendig et al. 2014). Up until 2009, surgical training was only possible at Queen Elizabeth Central Hospital (QECH) in Blantyre via the University of Malawi College of Medicine (COM) and through Fellowship programmes of the College of Surgeons of East Central and Southern Africa (COSECSA).

### Possible through COSECSA

It was possible to establish the postgraduate surgical training programme at KCH relatively quickly after the institutional collaboration was started with Haukeland University Hospital (HUH), despite a lack of a formal affiliation to a local university. Through support of KCH with academic medical staff, infrastructure and surgical equipment from HUH, The University of North Carolina (UNC) and other international partners, capacity was gradually built up in orthopaedic trauma surgery. This was possible with funding from the Norwegian government through the Royal Norwegian Embassy in Malawi and “Fredskorpset” (FK). The presence, since 1999, of a regional accrediting body for training of surgeons, The College of Surgeons of East Central and Southern Africa (COSECSA) made possible formal accreditation of KCH as a training site with minimal bureaucracy in 2009, once the COSECSA requirements for staff and infrastructure were met and a site visit confirmed conditions.

### From single site collaboration to national training programme: Norhed

Over time this new training programme at KCH has become sufficiently strong to match the existing training programme at Queen Elizabeth Central Hospital in Blantyre, under the University of Malawi College of Medicine (COM). For the sustainability of both training programmes the next logical step was to develop them into a national coordinated training programme for surgery under the College of Medicine. In 2013 a grant was secured from the Norwegian Agency for Development Cooperation (Norad), under the Norwegian Programme for Capacity Development in Higher Education and Research (Norhed), for COM to collaborate with the University of Bergen and HUH to develop this programme further. The goal is to train a critical mass of Malawian surgeons as future trainers and researchers, as well as to strengthen the academic qualifications of key COM surgery staff over the next 5 years.

### Current and potential impact

**Fig. 2:**
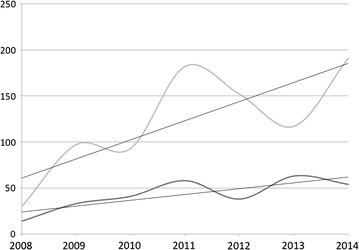
The rise in the use of limb saving surgery at KCH. The *top curve* shows the number of soft tissue procedures (debridements, flaps etc.) done for fractures per year, the *bottom curve* the number of external fixations for open fractures. The *straight lines* are the linear regression trend lines [external fixation: R^2^ 0.652, F(1,5) = 9.369, p = 0.028. Soft tissue procedures: R^2^ 0.630, F(1,5) = 8.508, p = 0.033]

**Fig. 3:**
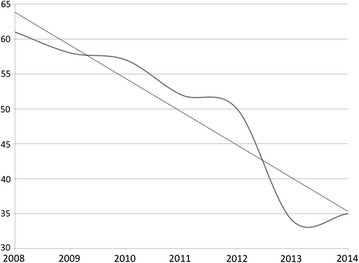
The number of amputations done at KCH was almost halved from 61 in 2008 to 35 in 2014. The *curve* represents the observed number of amputations done. The *straight line* is the linear regression trend line [R^2^ 0.880, F(1,5) = 36.745, p = 0.002]

**Fig. 4:**
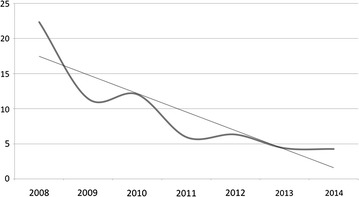
Nearly a quarter of all orthopaedic operations at KCH in 2008 were amputations. In 2014 this had fallen to under 5 %. The straight line represents the linear regression curve estimate confirming a clear reduction in the amputation rate [R^2^ 0.779, F(1,5) = 17.606, p = 0.009]

In orthopaedic trauma care the impact of the training programme at KCH can be seen in a year-by-year increase in limb saving surgery, such as external fixation and active surgical debridement of open fractures (Fig. 2), and a corresponding decrease in the rate of amputations done in the same time period (Fig. 3) as registered in the theatre log book at KCH. If the amputation rate is seen as a proportion of all orthopaedic surgery done at KCH the impact on current practice becomes even more striking (Fig. 4). In 2008 22 % of all orthopaedic operations at KCH were amputations. In 2014 this had fallen to 4 %. Significant regression equations for all these trends were found. (Increasing use of external fixation: R^2^ 0.652, F(1,5) = 9.369, p = 0.028. Increasing use of debridement: R^2^ 0.630, F(1,5) = 8.508, p = 0.033. Reduction of number of amputations: R^2^ 0.880, F(1,5) = 36.745, p = 0.002. Decrease in amputations as percentage of orthopaedic operations: R^2^ 0.779, F(1,5) = 17.606, p = 0.009).

The young doctors training in their home country of Malawi, are training as surgeons in the environment they are expected to work in as future consultants, and are exposed daily to the local panorama of disease and the suffering of the underserved poor. They will therefore be considerably better equipped to meet the challenges of orthopaedic practice in Malawi than a surgeon trained in a high-income country. The larger this group of locally trained specialist doctors gets, the stronger their voice for change. Short term exposure to well functioning health facilities abroad is still helpful, however, to learn modern techniques and to set their sights on how to improve the local health system. International institutional collaborations create a growing network that generates support and enables such short-term international training fellowships. Currently the surgical training programme in Malawi is collaborating with the Christian Medical College in Vellore, India, and the School of Medicine, Flinders University, Adelaide, Australia, to provide these short-term fellowships for Malawian orthopaedic surgery trainees.

### Serving the districts through strengthening of the centre

In low income countries, with limited human resources for health, the temptation to break up a growing group of new specialists in tertiary centres and send them to the districts, “where people need them the most”, should be resisted in the short term. It is often perceived as a failure that most specialists in a low-income country are in the largest cities. However, it is not necessarily so. Spreading highly specialized surgeons to many district hospitals where there is little equipment will cost more and only frustrate the surgeons who will not be able to use their training fully. Until sufficient numbers of physician surgeons can be trained, the districts are better served by task shifting to clinical officers or equivalent as well as improving infrastructure and surgical supplies for basic surgical care. Supporting, or helping to establish, teaching hospitals, enables them to continue producing new specialists for the future. They can be made more sustainable by offering paying services to the richer portion of the population with income generated from these services subsidising care for the poor, improving working conditions, and supporting infrastructure maintenance.

Resources invested in trauma care in LMICs with few available surgical specialists are better concentrated to show measurable effect. By establishing short stay trauma centres in centrally placed hospitals the underserved rural poor can also get access to modern trauma care. It is cheaper to transport a patient with a fractured femur from a district hospital to the trauma centre to get it fixed by a qualified surgeon than to build up fully functional trauma hospitals in all districts. Patients can be transferred back to the district hospital within a day or two where staff is qualified to carry out postop rehabilitation. This model secures equitable trauma treatment for as many as possible, avoids overcrowding of the central hospitals and helps the patients and their caretakers by letting them recover near home.

### International institutional collaboration

In our opinion, high income countries would get considerably more measurable results from their health sector support in LMICs by stimulating strong institutions in their own countries to partner, on a long term basis, with teaching hospitals in LMIC to strengthen these institutions and train more specialists in their own country. These institutional collaborations, however, need to be funded by agencies in the high income country so the supporting institution can be compensated for staff sent to their sister institution, and sufficient funds provided to run training activities. In any international collaboration it is also absolutely necessary for sustaining a training programme, or trauma centre, that sufficient funding is available for infrastructure and equipment improvement. Without this the institution cannot reach its potential, even with an on-going training programme, and there will be a higher risk of attrition of trainees and surgeons if they do not have the facilities necessary to do what they were trained for.

## Conclusions

Establishing a surgery training programme at Kamuzu Central Hospital in Malawi has dramatically improved outcomes for trauma victims. Long-term institutional collaboration in the training of surgeons in low-income countries is a sustainable and up-scalable model with great potential to reduce mortality and prevent disability in young people. Despite the obvious and necessary focus on the rural poor in low income countries, stakeholders must start to see the value of strengthening teaching hospitals to sustainably meet the growing burden of trauma and surgical disease.

## Methods

Annual operating data from Kamuzu Central Hospital’s Main Operating Theatre log book for the years 2008–2014 was collected. Observed annual numbers were presented as graphs for easy visualization. Linear regression curve estimations were calculated using IBM SPSS Statistics version 22 and plotted as trend lines on the graphs.

## References

[CR1] Crisp N, Chen L (2014). Global supply of health professionals. N Engl J Med.

[CR2] Gosselin RA, Heitto M, Zirkle L (2009). Cost-effectiveness of replacing skeletal traction by interlocked intramedullary nailing for femoral shaft fractures in a provincial trauma hospital in Cambodia. Int Orthop.

[CR3] Grimes CE, Henry JA, Maraka J, Mkandawire NC, Cotton M (2014). Cost-effectiveness of surgery in low- and middle-income countries: a systematic review. World J Surg.

[CR4] Hagander LE, Hughes CD, Nash K, Ganjawalla K, Linden A, Martins Y, Casey KM, Meara JG (2013). Surgeon migration between developing countries and the United States: train, retain, and gain from brain drain. World J Surg.

[CR5] Kendig C, Tyson A, Young S, Mabedi C, Cairns B, Charles A (2014). The effect of a new surgery residency program on case volume and case complexity in a sub-saharan african hospital. J Surg Educ.

[CR6] Kobusingye O, Guwatudde D, Lett R (2001). Injury patterns in rural and urban Uganda. Inj Prev.

[CR7] Patton GC, Coffey C, Sawyer SM, Viner RM, Haller DM, Bose K, Vos T, Ferguson J, Mathers CD (2009). Global patterns of mortality in young people: a systematic analysis of population health data. Lancet.

[CR8] Peden M (2004). World report on road traffic injury prevention. World report on road traffic injury prevention.

[CR9] WHO (2009). Global status report on road safety: time for action.

[CR10] WHO (2015). Global status report on road safety 2015.

